# Correlation of Surface Toll-Like Receptor 9 Expression with IL-17 Production in Neutrophils during Septic Peritonitis in Mice Induced by* E. coli*

**DOI:** 10.1155/2016/3296307

**Published:** 2016-02-25

**Authors:** Yunjia Ren, Li Hua, Xiuping Meng, Yue Xiao, Xu Hao, Sheng Guo, Peiyan Zhao, Luowei Wang, Boqi Dong, Yongli Yu, Liying Wang

**Affiliations:** ^1^Department of Molecular Biology in College of Basic Medical Sciences and Institute of Pediatrics in First Hospital, Jilin University, Changchun 130021, China; ^2^Department of Endodontics, School and Hospital of Stomatology, Jilin University, Changchun 130021, China; ^3^Department of Immunology in College of Basic Medical Sciences, Jilin University, Changchun 130021, China

## Abstract

IL-17 is a proinflammatory cytokine produced by various immune cells. Polymorphonuclear neutrophils (PMNs) are the first line of defense in bacterial infection and express surface Toll-like receptor 9 (sTLR9). To study the relationship of sTLR9 and IL-17 in PMNs during bacterial infection, we infected mice with* E. coli* intraperitoneally to establish a septic peritonitis model for studying the PMNs response in peritoneal cavity. We found that PMNs and some of “giant cells” were massively accumulated in the peritoneal cavity of mice with fatal septic peritonitis induced by* E. coli*. Kinetically, the CD11b^+^ PMNs were increased from 20–40% at 18 hours to >80% at 72 hours after infection. After* E. coli* infection, sTLR9 expression on CD11b^+^ and CD11b^−^ PMNs and macrophages in the PLCs were increased at early stage and deceased at late stage; IL-17 expression was also increased in CD11b^+^ PMNs, CD11b^−^ PMNs, macrophages, and CD3^+^ T cells. Using experiments of* in vitro* blockage, qRT-PCR and cell sorting, we confirmed that PMNs in the PLCs did increase their IL-17 expression during* E. coli *infection. Interestingly, sTLR9^−^CD11b^+^Ly6G^+^ PMNs, not sTLR9^+^CD11b^+^Ly6G^+^ PMNs, were found to be able to increase their IL-17 expression. Together, the data may help understand novel roles of PMNs in septic peritonitis.

## 1. Introduction

Sepsis affects approximately 700,000 people annually and accounts for about 210,000 deaths per year in the US. Its incidence is rising at rates between 1.5% and 8% per year, despite continuous progress in the development of antimicrobial therapeutics and supportive cares [1]. Most of the sepsis is caused by bacteria and bacteria of abdominal origin contribute to the second major reason for sepsis. This type of sepsis is designated as abdominal sepsis or septic peritonitis. The septic peritonitis is the host's systemic inflammatory response to the bacteria, initiated by the pathogen associated molecule patterns (PAMPs) including lipopolysaccharide and lipid A from Gram-negative bacteria and lipoteichoic acid and peptidoglycan from Gram-positive bacteria [2]. The inflammatory initiation leads to the release of chemokines, such as interleukin-8 (IL-8), and proinflammatory cytokines, such as tumor necrosis factor-*α* (TNF-*α*), IL-1, IL-6, and IL-12, in a massive amount. The cytokines, if not appropriately controlled, may severely impair the functions of vital organs or systems, resulting in death [2]. Data from the CIAOW study (complicated intra-abdominal infections worldwide observational study) showed that the overall mortality rate was 10.5% in the patients with septic peritonitis in a worldwide context [3].

Septic peritonitis is characterized by a massive infiltration of neutrophils into the peritoneum in response to bacterial invasion, where they are activated and act as a first line of defense against the bacteria [4]. Morphologically, the mature neutrophils are unique among the other white blood cells by their lobulated nucleus, which inspired the renaming of them as polymorphonuclear neutrophils (PMNs) [5]. Based on cytokine production, macrophage activation, expression of Toll-like receptor (TLR), and surface antigen expression, murine PMNs have been classified into three types [6]: normal PMN (PMN-N), PMN-I, and PMN-II. PMN-I produces IL-12/CCL3, activates macrophages in a classical way, expresses TLR2/TLR4/TLR5/TLR8, and has a CD49d^+^CD11b^−^ phenotype. PMN-II produces IL-10/CCL2, activates macrophages in an alternative manner, expresses TLR2/TLR4/TLR7/TLR9, and possesses a CD49d^−^CD11b^+^ phenotype. PMN-N rarely produces cytokine/chemokine, displays no effect on macrophage activation, and expresses TLR2/TLR4/TLR9, with CD49d^−^CD11b^−^ phenotypes. The PMN-N may convert to PMN-I or PMN-II in response to bacterial infections [6]. Recently, macrophages have been reported to cooperate with neutrophils by promoting extravasation and activation of PMNs, even their clearance of bacteria [7].

Accumulated evidence revealed that Toll-like receptors (TLRs) could play a fundamental role in induction of hyperinflammation and tissue damage in sepsis. TLRs are pattern recognition receptors (PRRs) that sense microbial invasion and initiate innate immune responses. Positioned at the cell surface, TLR4 is essential for sensing lipopolysaccharide of Gram-negative bacteria and TLR2 is involved in the recognition of a large panel of microbial ligands, while TLR5 recognizes flagellin. Endosomal TLR3, TLR7, TLR8, and TLR9 are specialized in the sensing of nucleic acids produced notably during viral infections or during bacterial infections [8]. Conventionally, TLR9 is thought to sense microbial DNA in endolysosomes and not at the cell surface. Recently, it has been found that TLR9 is expressed on the surface of human and mouse PMNs, that the surface TLR9 (sTLR9) senses DNA in PMNs, and that sTLR9 expressing PMNs (sTLR9^+^ PMNs) have roles in inducing rapid inflammation [9]. Human and mouse PMNs spontaneously express sTLR9, and the sTLR9 expression is upregulated in response to PMN stimulation [10]. In front of TLR9 ligands, sTLR9^+^ PMNs could be activated, leading to cytokine secretion and CD11b upregulation [10]. Notably, TLR9 stimulation was demonstrated to be detrimental in mice with bacterial sepsis. TLR9^−/−^ mice exhibited lower serum inflammatory cytokine levels, higher bacterial clearance, and greater survival after experimental peritonitis induced by cecal ligation and puncture (CLP). A single injection of TLR9 antagonist protected the wide type mice, even when administered as late as 12 hours after CLP [11]. It has been proposed that, during infection, sTLR9^+^ PMNs could be involved in the detrimental effect by releasing massive proinflammatory cytokines, including IL-6 and TNF-*α*, and possibly other cytokines in response to PAMPs [12, 13].

Interleukin-17 (IL-17) is a cytokine family that signatures T helper 17 (Th17) cell subset and contains six members (IL-17A to IL-17F), among which IL-17A is considered as one of the major proinflammatory cytokines mediating the innate and adaptive immune responses against bacterial infections [14]. In addition to Th17 cells, *γδ* T cells, innate lymphoid cells (ILCs), mast cells, PMNs, and macrophages are also IL-17 producing cells [14]. Notably, innate immune cell-derived IL-17 constitutes a major element in the immune response against infectious agents by recruiting PMNs to the sites of infections and by inducing the production of antimicrobial peptides, CXC chemokines, and granulocyte colony stimulating factor (G-CSF) [15, 16]. In bacterial infection, PMNs act to produce increased IL-17, which in turn recruits more PMNs to join the fighting against invaded bacteria, resulting in increased production of innate immune cell-derived IL-17 [16, 17]. IL-17 was found to work in an IL-23-IL-17 axis which was critical for the survival of the host infected with bacteria [18]. In the axis, tissue-infiltrating PMNs could be the main source of IL-23 [19] which induces production of IL-17 from macrophages [20].

In the present study, we kinetically observed the infiltration of PMNs and expression of sTLR9 and IL-17 on/in the PMNs in the peritoneal lavage cells (PLCs) of mice intraperitoneally infected with various doses of* Escherichia coli* (*E. coli*), aiming to find the correlation of the expression of sTLR9 and IL-17 on/in the PMNs during the development of bacterial septic peritonitis. The achieved results could provide a basis for further investigation on the roles of sTLR9 expressing PMNs and IL-17 producing PMNs in bacterium caused septic peritonitis.

## 2. Materials and Methods

### 2.1. Antibodies and Reagents

The monoclonal antibodies of APC-conjugated rat anti-mouse CD45 antibody (561689), PE-conjugated rat anti-mouse CD14 antibody (561711), PE-conjugated rat anti-mouse CD11b antibody (561689), APC-Cy*™* 7-cojugated rat anti-mouse CD3 antibody (560590), and APC-conjugated rat anti-mouse Ly-6G antibody (560599) were purchased from BD bioscience. The FITC-conjugated active anti-TLR9 monoclonal antibody (26C593.2) was from Abcam; FITC-conjugated anti-mouse IL-17A antibody (506908) and PerCP-Cy*™* 5.5-conjugated rat anti-mouse IL-17A (3354955) antibody were from Biolegend. Trizol reagent (NC0301) was from Invitrogen. cDNA Synthesis Kit (Transgene biotech, I21021) and two-step SYBR green qPCR assays (Transgene biotech, G31227) were from Biotech.

### 2.2. *E. coli* Strain and Mice

*E. coli* strain of JM109 was recovered from lyophilized powder by being suspended into LB medium and then cultured on LB agar plate at 37°C overnight. The single colonies of* E. coli* on the plate were used as a group of original seed* E. coli*.

Female ICR mice were purchased from the Experimental Animal Center, Medical College of Norman Bethune, Jilin University, and maintained in microisolator cages under specific pathogen-free conditions. All the mice were used at 6 to 8 weeks of age. The experimental manipulation of mice was undertaken in accordance with the National Institute of Health Guide for the Care and Use of Laboratory Animals, with the approval of the Scientific Investigation Board of Science & Technology of Jilin Province.

### 2.3. Preparation of* E. coli* Culture

To prepare* E. coli* culture, each single colony of* E. coli* was picked up and cultured in 5 mL LB medium at 37°C with shaking at 200 rpm. When the OD value (*A*_600_) of the culture reached 1.4,* E. coli* was harvested from the culture by centrifugation and mixed with 20% glycerol solution. The resultant glycerol* E. coli* was stored at −20°C as working* E. coli* seeds. The working seed* E. coli *were seeded into LB medium and cultured at 37°C till the OD value reached 0.7 followed by harvesting the* E. coli* pellet after centrifugation at 4000 g for 5 min. The* E. coli* pellet was diluted in a serial tenfold, plated on an eosin methylene blue agar plate, and then cultured at 37°C for 14 hours. The colony-forming units (CFUs) of the* E. coli* culture were calculated and determined as approximately 0.8 × 10^8^ CFUs/mL. For animal experiment use, the* E. coli *pellet was washed twice using sterile 0.9% NaCl (saline) and then resuspended in 1.0 mL saline containing 0.4, 0.8, 1.6, and 2.4 × 10^8^ CFUs of* E. coli*, respectively, and was ready to use for infecting mice.

### 2.4. Induction of Bacterial Septic Peritonitis and Preparation of PLCs

For inducing bacterial septic peritonitis, mice were injected intraperitoneally (i.p.) with 1.0 mL of* E. coli* preparations containing 0.4, 0.8, 1.6, and 2.4 × 10^8^ CFUs/mL in sterile saline, respectively. The saline injection was used as control. The survival of the mice was monitored every 4 hours for 4 days.

To harvest the peritoneal lavage cells (PLCs) from mice either infected with* E. coli* or injected with saline, the mice (*n* ≥ 3 per group) were euthanized at defined time points with 50 mg/kg pentobarbital sodium followed by washing peritoneal cavities using 6 mL per mouse of ice-cold phosphate-buffered saline (PBS). The peritoneal lavage fluid was centrifuged at 750 g for 5 minutes at 4°C for harvesting the PLCs. The PLCs were resuspended in cold PBS for further use.

### 2.5. Cell Culture and Treatment

PLCs were harvested from the naïve mice and maintained in RPMI 1640 supplemented with 10% (V/V) fetal bovine serum (FBS) (GIBCO) and antibiotics (100 IU of penicillin/mL and 100 IU of streptomycin/mL). In experiments using viable bacteria, antibiotics were not added to the culture medium during the isolation, washing, or subsequent culturing period. No bacterial contamination was observed in PLCs cultured in the absence of antibiotics. Cells were counted and then plated in 24-well cell culture plates (Costar, Cambridge, MA) at an approximate density of 1 × 10^6^ cells/well. The PLCs were cocultured with* E. coli* at 1 × 10^5^ or 1 × 10^6^ CFUs/well or saline as a vehicle control for 14 hours, and then they were cultured with BFA for another 4 hours, in a 5% CO_2_ humidified incubator at 37°C. The PLCs were collected and stained with FITC-labeled anti-IL-17 mAb, followed by flow cytometry analysis to detect the expression of IL-17.

### 2.6. Cell Counting

To count the numbers of cells in each peritoneal lavage sample, the samples were spin down for harvesting the cell pellets. The cell pellets were smeared on slides and then stained using hematoxylin-eosin (HE) followed by counting the cell numbers on hemocytometer (Beckman Coulter, Fullerton, CA) and taking photos of the cells.

### 2.7. Flow Cytometry

For surface staining, the PLCs were stained with fluorescence-conjugated mAbs against CD45, CD11b, and sTLR9, respectively, for 30 minutes at room temperature in the dark followed by washing twice with PBS. For IL-17 intracellular staining, the PLCs were surface stained as described above and then fixed with 4% paraformaldehyde and permeabilized with 0.1% saponin followed by staining with FITC-conjugated anti-IL17A monoclonal antibody. All stained cells were analyzed by flow cytometer FACSCalibur (BD) and CytoFLEX (Beckman Coulter). Live cells were carefully gated by forward and side scattering. Data were analyzed with FlowJo software (FlowJo 7.6.1).

### 2.8. Cell Sorting

The PLCs were harvested from mice (*n* ≥ 6 per group) either infected with* E. coli* or injected with saline. The PLCs were stained with fluorescence-conjugated mAbs against CD14, CD11b, and CD3 for 30 minutes at room temperature in the dark, followed by washing twice with PBS. The stained cells were sorted using BD FACS Aria II by the methods described in Figure 5(g). The neutrophils were sorted by selection for CD3^−^CD14^−^CD11b^+^ cells, and T cells were sorted by selection for CD3^+^ cells, according to the manufacturer's recommendations. T cells (defined as CD3^+^ cells with a purity >83% of living cells) and PMNs (defined as CD3^−^CD14^−^CD11b^+^ cells with a purity >96.7% of living cells) were obtained.

### 2.9. qRT-PCR

Total RNA was isolated from the PLCs with Trizol reagent and reverse-transcribed using cDNA Synthesis Kit. Quantitative real-time PCR (qRT-PCR) was performed using two-step SYBR green qPCR assays and the target mRNAs were identified by the specific primers as follows: IL-17A, forward: 5′-AAGGCAGCAGCGATCATCCCT; reverse: 3′-TCTTCATTGCGGTGGAGAGTCC; GAPDH, forward: 5′-ATCACCATCTTCCAGGAGCGA; reverse: 3′-TCTCGTGGTTCACACCCATCA. The data were acquired using the Step One*™* real-time PCR system (Applied Biosystems). The procedure of the target mRNA amplification was as follows: 1 cycle at 95°C (30 seconds) followed by 40 cycles at 95°C (5 seconds) and 64°C (31 seconds). Each assay plate included negative controls with no template. The relative amount of gene expression was calculated according to the formula 2^−ΔΔCt^, in which ΔCt = [Ct(gene) − Ct(GAPDH)] and Ct is the crossing threshold value returned by the PCR instrument for every gene amplification.

### 2.10. Statistical Analysis

Comparisons between groups were conducted using analysis of Student's *t*-tests. Survival curves of mice were estimated using the Kaplan-Meier method and compared using the log-rank test. We considered the resulting *P* values of less than 0.05 (95% CI) to be statistically significant. Statistics were analyzed using GraphPad Prism 5.0 for Windows (San Diego, CA).

## 3. Results

### 3.1. The Establishment of* E. coli* Induced Septic Peritonitis in Mice

**Figure 1:**
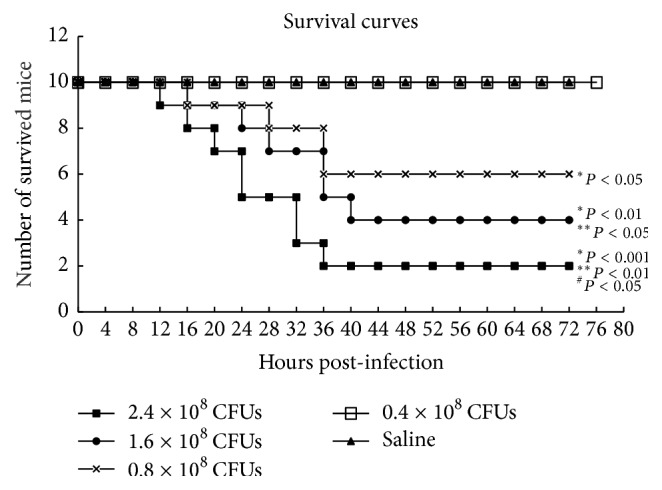
Survival curves of mice with septic peritonitis induced by* E. coli*. ICR mice were infected intraperitoneally with* E. coli* at different doses for inducing septic peritonitis. Saline-injected mice were as controls. The survival of the mice was counted. ^*∗*^Compared to control mice; ^*∗∗*^compared to mice infected with 0.4 × 10^8^ CFUs of* E. coli*; ^#^compared to mice infected with 1.6 × 10^8^ CFUs of* E. coli*.

To understand how neutrophils contribute to the development of sepsis, we first established a murine model of septic peritonitis induced by* Escherichia coli* (*E. coli*). The female ICR mice, 10 in each group, were intraperitoneally injected with 2.4 × 10^8^, 1.6 × 10^8^, 0.8 × 10^8^, and 0.4 × 10^8^ colony forming units (CFUs) of* E. coli*, respectively, and then monitored for their physical conditions and survival. Around 6 hours after the infection, all of the 10 mice received 2.4 × 10^8^ CFUs of* E. coli*, 8 out of the 10 mice received 1.6 × 10^8^ CFUs of* E. coli*, and 5 out of the 10 mice that received 0.8 × 10^8^ CFUs of* E. coli* began to exhibit multiple neurological symptoms, including dispirited behavior, staggered gait, and trembling, but these phenomena were not present in all of the 10 mice that received 0.4 × 10^8^ CFUs of* E. coli* or saline. Around 18 hours after the infection, the infected mice began to die. Around 48 hours, the infected mice underwent a massive death, especially in those that received high infectious dose of* E. coli*. By 72 hours after the infection, 80%, 60%, and 40% of the mice infected with 2.4, 1.6, and 0.8 × 10^8^ CFUs of* E. coli*, respectively, died and yet 100% of the mice which were infected with 0.4 × 10^8^ CFUs of* E. coli* or received saline survived (Figure 1). The results indicated that the infection with* E. coli* at 1.6 × 10^8^ CFUs and 2.4 × 10^8^ CFUs could induce septic peritonitis in ICR mice.

### 3.2. The Infiltration and Morphology of Neutrophils in PLCs of Mice with Septic Peritonitis Induced by* E. coli*

**Figure 2:**
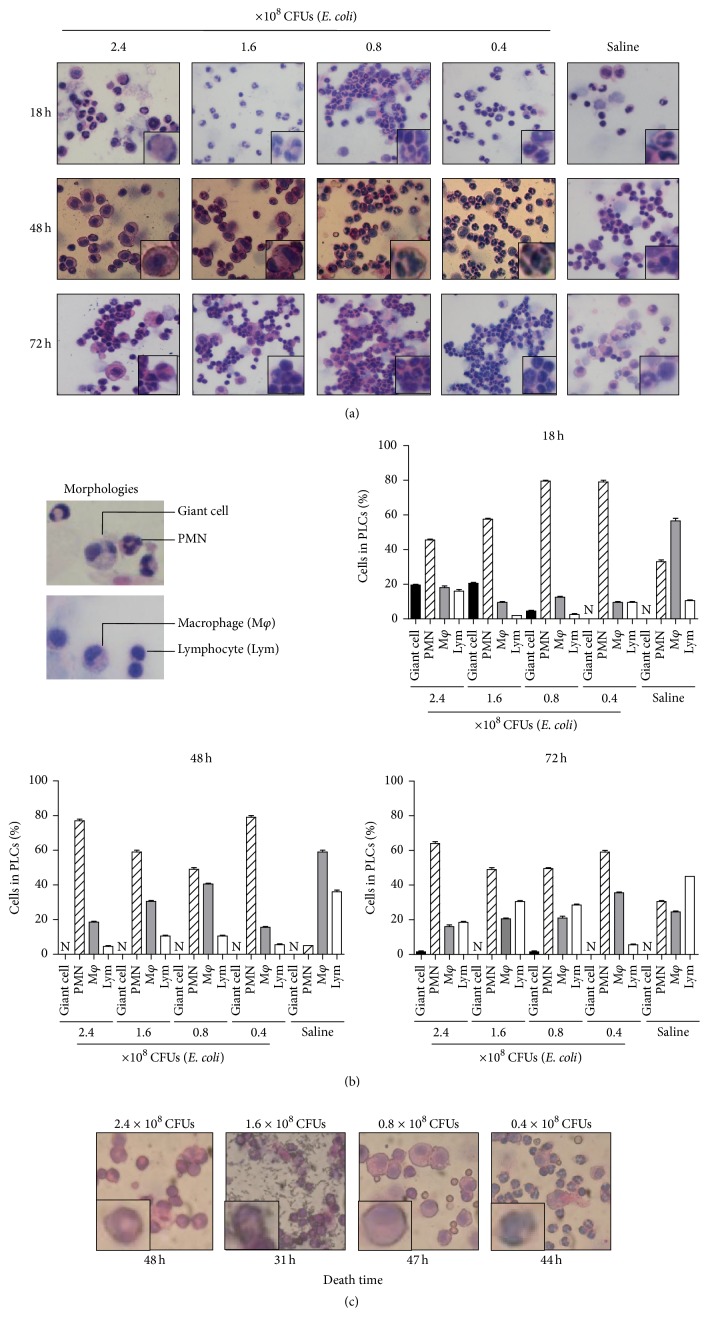
Morphologies of peritoneal lavage cells (PLCs) from mice infected intraperitoneally with* E. coli*. ICR mice were infected intraperitoneally with 3 doses of* E. coli* or received saline and then sacrificed at 18, 48, and 72 hours, respectively, for harvesting their PLCs. The PLCs were stained with H&E dye followed by taking photos and counting cell numbers under a microscope. (a) Photos of PLCs from killed mice. (b) The cell counts based on the PLC morphologies. (c) Photos of PLCs from dying mice. Each group is composed of 6 mice.

Since the polymorphonuclear cells (PMNs) are documented as the first innate immune cells recruited at inflammation sites and play a central role in host defense [21], we next collected the peritoneal lavage cells (PLCs) of the mice at 18, 48, and 72 hours after infection, respectively, for observing the infiltration and morphology of the PMNs in the PLCs. The collected PLCs were fixed on glass slides, stained with H&E dye, and photographed. A massive infiltration of PMNs was observed in the PLCs from the infected mice. At 18 hours after infection, in the PLCs of the mice infected with 1.6 and 2.4 × 10^8^ CFUs of* E. coli*, the majority of the cells with the size of 0.2~0.4 micrometer were PMNs with a typical lobulated nucleus. Interestingly, there occurred a portion of giant cells, with the size of nearly 1.0~1.6 micrometer, characterized by lobulated nucleus that was squeezed to the marginal zone of the inner cell membrane. However, at 48 or 72 hours, the giant cells disappeared in the PLCs of the mice infected with 1.6 and 2.4 × 10^8^ CFUs of* E. coli* (Figure 2(a)). Morphologically, there are four types of cells in the PLCs, including PMNs, macrophages, lymphocytes, and the giant cells (Figure 2(b)). When counting the cells, we found that at 18, 48, and 72 hours, PMNs constituted 50%–80% and macrophages constituted 20%–40% of the PLCs from the mice infected with 4 doses of* E. coli*, respectively, compared with nearly 30% for PMNs and 60% for macrophages in the PLCs from the mice that received saline. At 18 hours, interestingly, there were about 20% of the giant cells in the PLCs of the mice infected with 1.6 and 2.4 × 10^8^ CFUs of* E. coli* (Figure 2(b), upper right). Noticeably, when the mice died of the infection, we collected the PLCs immediately and observed and found that plenty of bacteria coexisted with the PMNs in the PLCs of the mouse infected with 1.6 × 10^8^ CFUs of* E. coli *for 31 hours (Figure 2(c)). These results indicate that neutrophils can move to infected peritoneal cavity by infiltration and change their morphologies after engulfing bacteria possibly.

### 3.3. The Correlation of Neutrophil Numbers in PLCs with Severity of Septic Peritonitis in Mice Infected with* E. coli*

**Figure 3:**
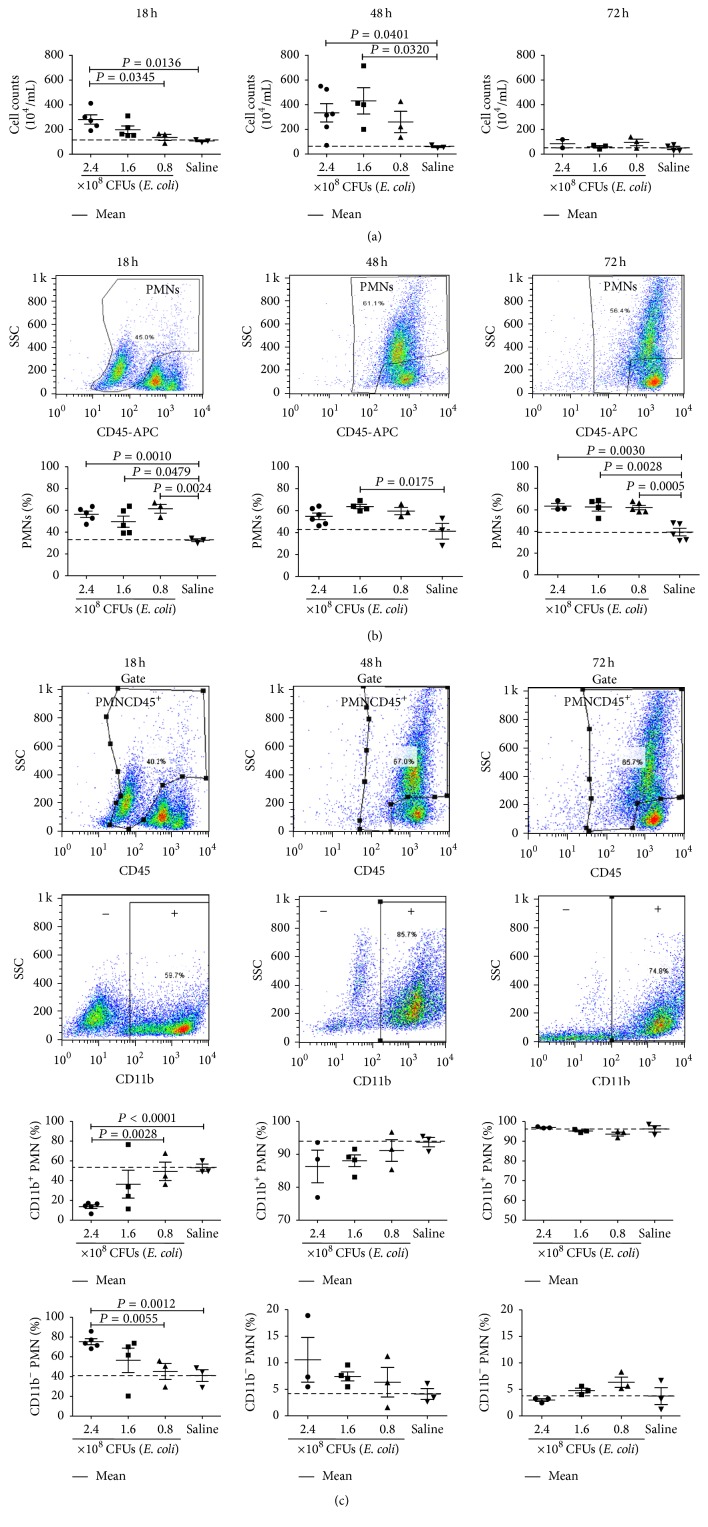
The numbers of PLCs and PMNs in mice infected intraperitoneally with* E. coli*. ICR mice were infected intraperitoneally with 3 doses of* E. coli* or received saline and sacrificed at 18, 48, and 72 hours to harvest their PLCs. The PLCs were counted immediately or stained with fluorescence-labeled mAbs of anti-CD45 and anti-CD11b followed by numerating with flow cytometry. (a) Numbers of PLCs. (b) Ratios of PMNs in PLCs. (c) Ratios of CD11b^+^ PMNs and CD11b^−^ PMNs in the PMNs. Each point represents the data from one mouse.

During septic peritonitis, a large number of PMNs were recruited to the peritoneal cavity of the infected mice. To find the correlation of the numbers of PMNs at various time points with the severity of the disease in mice infected with* E. coli*, the PLCs of the mice were collected at 18, 48, and 72 hours after infection, counted by hemocytometer or flow cytometry after staining with fluorescence-labeled mAb of anti-CD45 and anti-CD11b. At 18 hours, the numbers of PLCs were nearly 1 × 10^6^ cells/mL of peritoneal lavage fluid (PLF) in the mice received saline (control mice), significantly increased in the mice infected with 3 doses of* E. coli* in a dose dependent manner, and even reached nearly 3 × 10^6^ cells/mL of PLF in the mice infected with 2.4 × 10^8^ CFUs of* E. coli* (*P* < 0.05). At 48 hours, the numbers of PLCs were less than 1 × 10^6^ cells/mL of PLF in control mice and significantly increased to over 3 × 10^6^ cells/mL of PLF in the mice infected with three doses of* E. coli* and even to about 4 × 10^6^ cells/mL of PLF in the mice infected with 1.6 and 2.4 × 10^8^ CFUs of* E. coli* (*P* < 0.05). At 72 hours, the numbers of PLCs were decreased to 0.5 × 10^6^ cells/mL of PLF in control mice and decreased to nearly 0.5 × 10^6^ cells/mL of PLF in the mice infected with three doses of* E. coli* (Figure 3(a)). In the PLCs from the infected mice, most of the cells were CD45^+^ nucleated leukocytes, representing PMNs, macrophages, and lymphocytes, respectively. At 18 hours, the PMNs, macrophages, and lymphocytes were displayed on the left, middle, and right of the histogram, respectively, sequentially based on their nuclear structure and fluorescence intensity. At 48 and 72 hours, the PMNs were found to be shifted to the right upper quadrant, indicating that the PMNs were with more ovulated nuclei and expressed elevated CD45. As shown in Figure 3(b), at 18 hours after infection, the CD45^+^ PMNs constituted 61.5%, 49.6%, and 56.4% of the PLCs in the mice infected with 0.8, 1.6, and 2.4 × 10^8^ CFUs of* E. coli*, respectively, compared with 32.5% in the control mice. At 48 hours, the CD45^+^ PMNs were increased to 59.5%, 63.7%, and 54.7% in the PLCs from the mice infected with 0.8, 1.6, and 2.4 × 10^8^ CFUs of* E. coli*, respectively, compared with 41.2% from the control mice. At 72 hours, the CD45^+^ PMNs constituted 62.2%, 62.8%, and 63.5% of the PLCs in the mice infected with 0.8, 1.6, and 2.4 × 10^8^ CFUs of* E. coli*, respectively, compared with 39.5% from the control mice. The PMNs were further detected as CD11b^+^CD45^+^ PMNs and CD11b^−^CD45^+^ PMNs, and their ratios in the PMNs were calculated. As shown in Figure 3(c), the CD11b^+^CD45^+^ PMNs constituted 49%, 36%, and 13% of the PMNs in the PLCs from the mice infected with 0.8, 1.6, and 2.4 × 10^8^ CFUs of* E. coli* for 18 hours, respectively. At 48 hours after the infection, the CD11b^+^CD45^+^ PMNs constituted 91%, 88%, and 86% in the mice infected with 0.8, 1.6, and 2.4 × 10^8^ CFUs of* E. coli*, respectively. At 72 hours after the infection, the CD11b^+^ PMNs reached around 96% in all infected mice. Oppositely, at 18 hours, the CD11b^−^ PMNs constituted 51%, 64%, and 87% of the PMNs in the PLCs from the mice infected with 0.8, 1.6, and 2.4 × 10^8^ CFUs of* E. coli*, respectively. At 48 hours after the infection, the CD11b^−^ PMNs constituted 9%, 12%, and 14% in the mice infected with 0.8, 1.6, and 2.4 × 10^8^ CFUs of* E. coli*, respectively. At 72 hours after the infection, the CD11b^−^ PMNs only accounted for around 4% in all infected mice (Figure 3(c)). The results indicated that the peritoneal infection of* E. coli* induced massive infiltration of CD45^+^ PMNs. Among the CD45^+^ PMNs, CD11b^+^ PMNs were increased with the progression of infection. In parallel, the CD11b^−^ PMNs were decreased. At 18 and 48 hours after infection, the ratios of CD11b^+^ PMNs or CD11b^−^ PMNs were correlated with the doses of* E. coli* negatively or positively.

### 3.4. The Expression of Surface TLR9 (sTLR9) on Neutrophils and Macrophages in PLCs with Development of Septic Peritonitis in Mice Infected with* E. coli*

**Figure 4:**
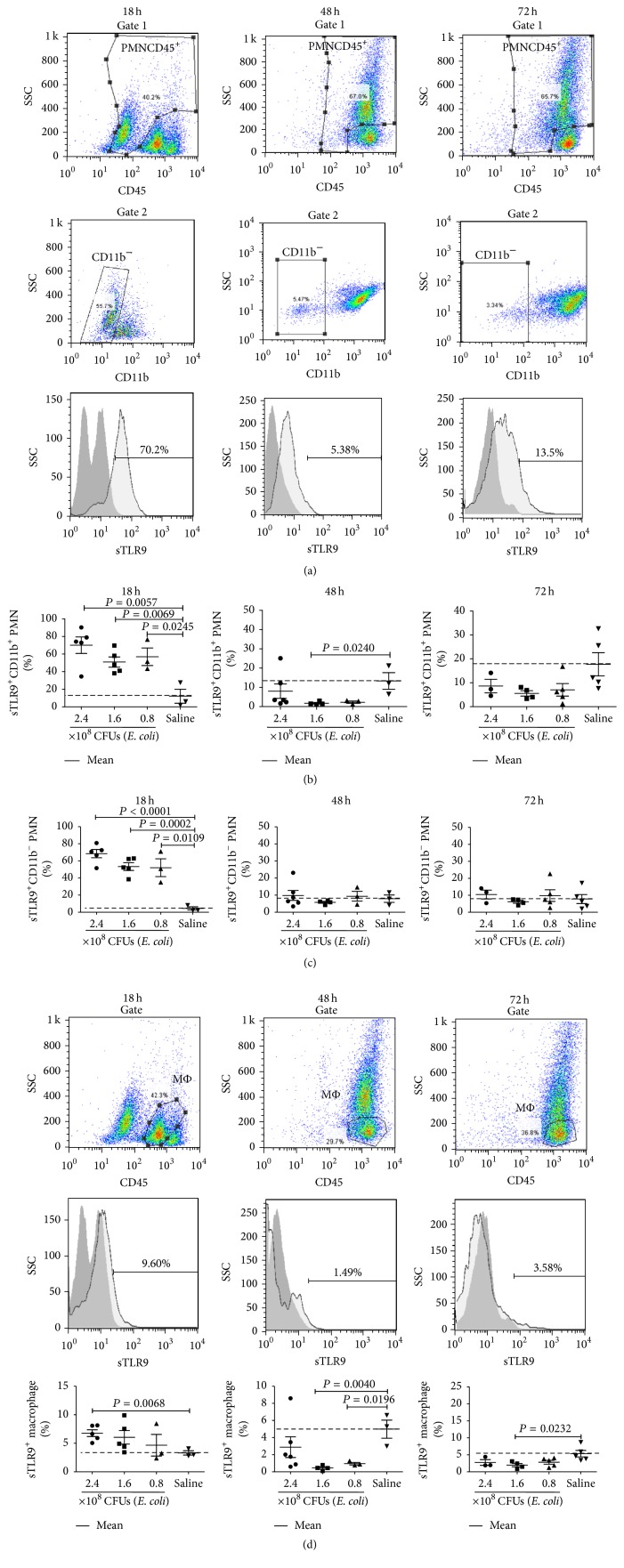
The ratios of sTLR9^+^ PMNs and macrophages in PLCs from mice infected intraperitoneally with* E. coli*. ICR mice were infected intraperitoneally with 3 doses of* E. coli* or received saline and sacrificed at 18, 48, and 72 hours to harvest their PLCs. The PLCs were stained with fluorescence-labeled mAb of anti-CD45, anti-CD11b, and anti-TLR9 followed by detection with flow cytometry. (a) Gates of the PMNs from the CD45^+^ PLCs followed by gating CD11b^+^ PMNs or CD11b^−^ PMNs from the PMNs. (b) Ratios of sTLR9^+^ PMNs in CD11b^+^ PMNs. (c) Ratios of sTLR9^+^ PMNs in CD11b^−^ PMNs. (d) Ratios of sTLR9^+^ macrophages. Each point represents the data from one mouse.

Since primary human and mouse blood neutrophils were reported to be able to express a functional sTLR9 [10], we tried to detect whether the expression of sTLR9 was different on CD11b^+^ PMNs and CD11b^−^ PMNs and correlates with development of septic peritonitis in mice. The PLCs collected from the mice which were infected with* E. coli* or received saline were gated for detecting the expression of sTLR9 on both CD11b^+^ and CD11b^−^ PMNs (Figure 4(a)). Results showed that, at 18 hours after infection, the ratios of sTLR9^+^CD11b^+^ PMNs were increased to 57%, 51%, and 70% of the CD11b^+^ PMNs in the mice infected with 0.8, 1.6, and 2.4 × 10^8^ CFUs of* E. coli*, respectively (*P* < 0.05), compared to 12% in the mice that received saline control (control mice). At 48 hours, the ratios of sTLR9^+^CD11b^+^ PMNs were decreased to 2.3%, 2.3%, (*P* < 0.05) and 8% in the infected mice, respectively, and less than 13% as in control mice. At 72 hours, the ratios of sTLR9^+^CD11b^+^ PMNs were 7%, 5.5%, and 9% in the infected mice, respectively, compared with 18% in control mice (Figure 4(b)). The ratios of sTLR9^+^CD11b^−^ PMNs constituted 52%, 53%, and 68.5% of the CD11b^−^ PMNs in the mice infected with 0.8, 1.6, and 2.4 × 10^8^ CFUs of* E. coli*, respectively, for 18 hours (*P* < 0.05), compared with 3.5% in control mice, sharply decreased to 9%, 6%, and 9.5% in the mice infected with 0.8, 1.6, and 2.4 × 10^8^ CFUs of* E. coli*, respectively, for 48 hours, compared with 8% in control mice, and reached 10%, 6%, and 10% in the mice infected with 0.8, 1.6, and 2.4 × 10^8^ CFUs of* E. coli*, respectively, for 72 hours, compared with 8% in control mice (Figure 4(c)). These results indicated that* E. coli* could significantly induce the occurrence of both of the sTLR9^+^CD11b^+^ PMNs and sTLR9^+^CD11b^−^ PMNs in the peritoneal cavity at early stage of infection. Considering the fact that macrophages are the inflammatory cells which overlapped with the PMNs in the inflammatory sites, we also observed sTLR9 expression on macrophages in the PLCs. The results showed that the ratios of sTLR9^+^ macrophages constituted 5%, 6%, and 6.7% (*P* < 0.05) of the macrophages in the mice infected with 0.8, 1.6, and 2.4 × 10^8^ CFUs of* E. coli*, respectively, for 18 hours, compared with 3% in control mice, decreased to 0.9% (*P* < 0.05), 0.45% (*P* < 0.05), and 3% in the mice infected with 0.8, 1.6, and 2.4 × 10^8^ CFUs of* E. coli*, respectively, for 48 hours, compared with 5% in control mice, and reached 4.8%, 2% (*P* < 0.05), and 2.8% in the mice infected with 0.8, 1.6, and 2.4 × 10^8^ CFUs of* E. coli*, respectively, for 72 hours, compared with 5.5% in control mice (Figure 4(d)). These results indicated that, unlike PMNs, the macrophages barely express increased sTLR9 during septic peritonitis.

### 3.5. Expression of Interleukin-17 (IL-17) in Neutrophils in PLCs from Mice with Septic Peritonitis Induced by* E. coli*

**Figure 5:**
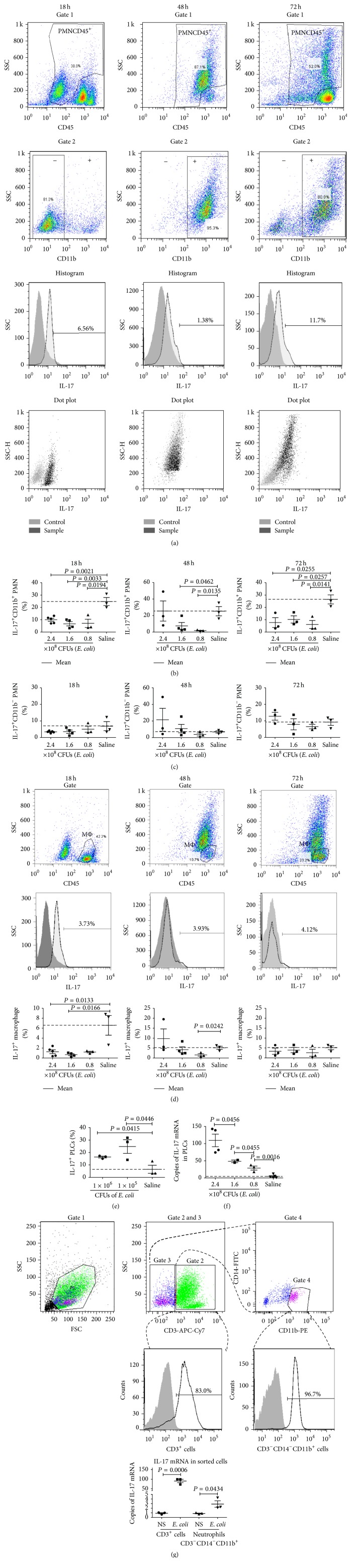
The ratios of IL-17^+^ PMNs and macrophages in PLCs from mice infected with* E. coli* intraperitoneally. ICR mice were infected with* E. coli* at 3 doses or received saline and sacrificed at 18, 48, and 72 hours to harvest their PLCs. The PLCs were stained with fluorescence-labeled mAb of anti-CD45, anti-CD11b, and anti-IL-17, followed by detection with flow cytometry or lysed to isolate total RNA for amplifying IL-17 mRNA by qPCR. (a) PMNs gated from the CD45^+^ PLCs (gate 1) and CD11b^+^ PMNs or CD11b^−^ PMNs from the gated PMNs (gate 2). (b) Ratios of IL-17^+^ PMNs in CD11b^+^ PMNs. (c) Ratios of IL-17^+^ PMNs in CD11b^−^ PMNs. (d) Ratios of IL-17^+^ macrophages. (e) Percentages of IL17^+^ PLCs in the PLCs cocultured with* E. coli*. (f) Copies of IL-17 mRNA in the PLCs. Each point represents the data from one mouse. (g) IL-17 mRNA expression in sorted CD3^+^ cells or CD3^−^CD14^−^CD11b^+^ neutrophils.

In recent years, primary human and mouse neutrophils have been found to be able to display autocrine IL-17 activity that probably contributes to the etiology of microbial and inflammatory diseases [17]. To find whether the different subtype neutrophil derived IL-17 was involved in the development of septic peritonitis, we detected IL-17 expression in the CD11b^+^ PMNs and CD11b^−^ PMNs (Figure 5(a)) of the PLCs collected from the mice which were infected with* E. coli* or received saline (control mice). It was found that the ratios of IL-17^+^CD11b^+^ PMNs constituted 7%, 8.5%, and 10% of the CD11b^+^ PMNs in PLCs of the mice infected with 0.8, 1.6, and 2.4 × 10^8^ CFUs of* E. coli*, respectively, for 18 hours (*P* < 0.05), compared with 25% in control mice, was 2% (*P* < 0.05), 7.5% (*P* < 0.05), and 25.5% in the mice infected with 0.8, 1.6, and 2.4 × 10^8^ CFUs of* E. coli*, respectively, for 48 hours, compared with 25% in control mice, and reached 6%, 10%, and 8% in the mice infected with 0.8, 1.6, and 2.4 × 10^8^ CFUs of* E. coli*, respectively, for 72 hours (*P* < 0.05), compared with 27% in control mice (Figure 5(b)). Noticeably, the saline injection seemed to stimulate neutrophils to express IL-17. To confirm this, the PLCs were collected from the mice injected with saline at 18, 48, or 72 hours after the injection, respectively, and stained with fluorescence-labeled mAb against CD45, CD11b, and IL-17, followed by detection using flow cytometry. The control mice in this experiment were not injected with saline. We found that saline stimulation tended to induce IL-17 expression (not statistically significant) with a big range of variation individually (data not shown). The ratios of the IL-17^+^CD11b^−^ PMNs were 5%, 4%, and 3% of the CD11b^−^ PMNs in the mice infected with 0.8, 1.6, and 2.4 × 10^8^ CFUs of* E. coli*, respectively, for 18 hours, compared with 7% in control mice; 3%, 11%, and 21.5% in the mice infected with 0.8, 1.6, and 2.4 × 10^8^ CFUs of* E. coli*, respectively, for 48 hours, compared with 7% in control mice; and 7%, 8%, and 13% in the mice infected with 0.8, 1.6, and 2.4 × 10^8^ CFUs of* E. coli*, respectively, for 72 hours, compared with 9% in control mice (Figure 5(c)). The results indicated that both of the CD11b^+^ PMNs and the CD11b^−^ PMNs could not increase IL-17 expression during the development of septic peritonitis in mice induced by* E. coli*. Also, as shown in Figure 5(d), macrophages could not increase the IL-17 expression in response to* E. coli*. Obviously, the results were in disagreement with the reports showing that PMNs could increase the production of IL-17 during bacterial infections. To validate the* in vivo* expression of IL17 in the PLCs, the PLCs pooled from the naïve mice were cocultured with* E. coli* at 1 × 10^5^ CFUs, 1 × 10^6^ CFUs or saline for 14 hours, and then with BFA for another 4 hours. The cultured cells were harvested, stained with FITC-labeled anti-IL-17 mAb, and detected by flow cytometry. As shown in Figure 5(e),* E. coli* could significantly increase IL-17^+^ PLCs of the naïve mice, indicating that* E. coli* could stimulate the PLCs to express IL-17. To clarify this, we set up an experiment to detect IL-17 mRNA expression in the PLCs from the mice infected with 0.8, 1.6, and 2.4 × 10^8^ CFUs of* E. coli *for 18 hours, respectively, or from the control mice and found that the IL-17 mRNA levels in the mice infected with 3 doses of* E. coli* were significantly elevated in a dose dependent manner (Figure 5(f)). Next we sorted CD3^+^ cells and CD3^−^CD14^−^CD11b^+^ cells from the PLCs of the mice which were infected with* E. coli* or received saline for 18 hours, detected their IL-17 mRNA expression by qRT-PCR, and found that IL-17 mRNA levels were significantly increased in both of the CD3^+^ T cells (*P* = 0.0006) and the CD3^−^CD14^−^CD11b^+^ cells (*P* = 0.0434) in the PLCs from the* E. coli* infected mice. Comparatively, the IL-17 mRNA levels in the CD3^+^ T cells were much higher than those in the CD3^−^CD14^−^CD11b^+^ neutrophils. The result indicated that both of T cells and PMNs were the IL-17 producers in the peritoneal cavity during the development of septic peritonitis (Figure 5(g)). The result suggests that the PLCs from the infected mice did express IL-17 and IL-17 in the PMNs might be secreted out during the development of septic peritonitis.

### 3.6. Correlation of sTLR9 Expression with IL-17 Production in Neutrophils at Early Stage of* E. coli* Induced Septic Peritonitis in Mice

**Figure 6:**
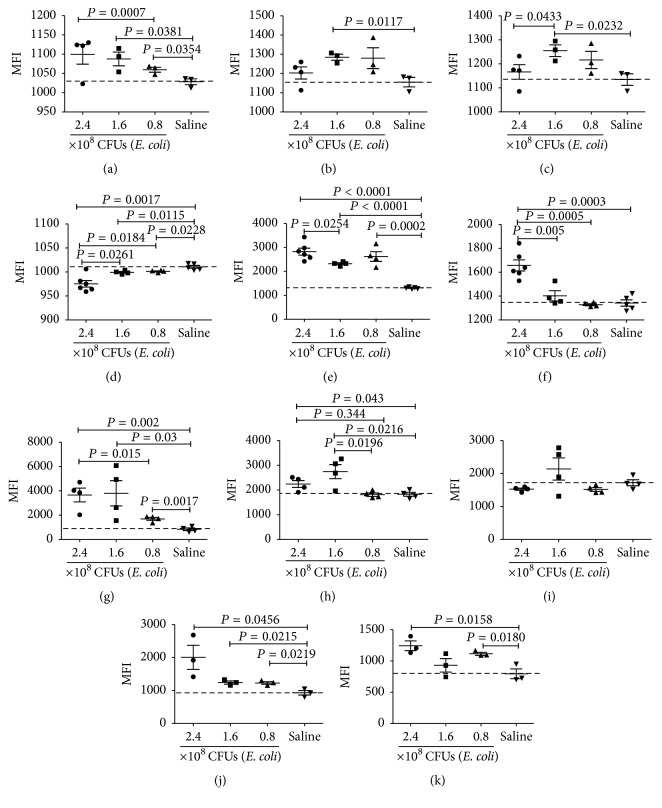
The expression of IL-17 and sTLR9 in/on PMNs, macrophages, and T cells in the PLCs from mice infected with* E. coli* intraperitoneally at early stage after infection. ICR mice which were infected with* E. coli* at 3 doses or received saline were sacrificed at 8 hours after infection for harvesting their PLCs. The PLCs were stained with fluorescence-labeled mAb of anti-CD45, anti-CD11b, anti-IL-17, anti-sTLR9, anti-CD3, and anti-Ly6G, followed by detection with flow cytometry. The expression levels of IL-17 and sTLR9 in various cells of the PLCs were indicated by mean fluorescence intensity (MFI). (a) IL-17 in CD11b^−^ PMNs. (b) IL-17 in CD11b^+^ PMNs. (c) IL-17 in macrophages. (d) sTLR9 on CD11b^−^ PMNs. (e) sTLR9 on CD11b^+^ PMNs. (f) sTLR9 on macrophages. (g) sTLR9 on CD11b^+^Ly6G^+^ PMNs. (h) IL-17 in CD11b^+^Ly6G^+^ PMNs. (i) IL-17 in sTLR9^+^CD11b^+^Ly6G^+^ PMNs. (j) IL-17 in CD3^+^ PLCs. (k) IL-17 in CD3^−^CD11b^+^ PMNs. Each point represents the data from one mouse.

To find the possible correlation between the production of IL-17 and the expression of sTLR9 in/on the PMNs and macrophages in peritoneal cavity of the mice during septic peritonitis, we harvested the PLCs from the mice infected with 0.8, 1.6, and 2.4 × 10^8^ CFUs of* E. coli*, respectively, at early stage of 8 hours after infection and detected their expression of IL-17 and sTLR9. The saline injected mice were as controls. The expression was represented by mean fluorescence intensity (MFI) to emphasize the increased expression of IL-17 and sTLR9 per cell. As shown in Figure 6, IL-17 expression was significantly upregulated in the CD11b^−^ PMNs from the infected mice and their levels were positively correlated with the doses of* E. coli* (Figure 6(a)), while the upregulated IL-17 expression was only observed in the CD11b^+^ PMNs or macrophages from the mice infected with 0.8 × 10^8^ CFUs and 1.6 × 10^8^ CFUs of* E. coli*. The highest dose of* E. coli* could not induce the highest levels of IL-17 in the CD11b^+^ PMNs or macrophages after infection for 8 hours (Figures 6(b) and 6(c)). This result reveals that IL-17 may be mainly derived from CD11b^−^ PMNs and secrete out earlier during bacterial infection in mice infected with high dose of* E. coli*. In parallel, we also tested the expression of sTLR9 on PMNs and macrophages and found that sTLR9 expression was significantly downregulated in the CD11b^−^ PMNs from the infected mice and the lowest downregulation happened in the CD11b^−^ PMNs from the mice infected with 2.4 × 10^8^ CFUs of* E. coli* (Figure 6(d)). In contrast, sTLR9 expression was significantly upregulated in the CD11b^+^ PMNs from the infected mice (Figure 6(e)). Interestingly, the significantly upregulated expression of sTLR9 was observed also in the macrophages from the mice infected with 2.4 × 10^8^ CFUs of* E. coli* (Figure 6(f)). To validate whether other types of cells in the PLCs also expressed IL-17 and sTLR9, the PLCs from mice infected with* E. coli* for 8 hours were stained with fluorescence-labeled mAbs against Ly6G, CD11b, IL-17, or TLR9 and then analyzed by flow cytometry. The results showed that* E. coli* infection significantly increased IL-17 expressing CD11b^+^Ly6G^+^ neutrophils (Figure 6(h)) as well as sTLR9 expressing CD11b^+^Ly6G^+^ neutrophils (Figure 6(g)) in a dose dependent manner but could not increase IL-17 expression in the sTLR9^+^CD11b^+^Ly6G^+^ neutrophils (Figure 6(i)) at the time point. In parallel, the PLCs were stained with fluorescence-labeled mAbs against CD3, CD11b, and IL-17 and analyzed. It was found that both CD3^+^ T cells (Figure 6(j)) in the PLCs and CD3^−^CD11b^+^ PMNs (Figure 6(k)) increased their IL-17 expression in response to the infection with* E. coli* for 8 hours. This result indicates that, at early stage, sTLR9 may be mainly expressed on CD11b^+^ PMNs in PLCs of the mice infected with* E. coli*, and with the infection progression, macrophages may become successors to replace the CD11b^+^ PMNs as the major sTLR9^+^ cells.

## 4. Discussion

In this study, we tried to investigate the correlation of TLR9 expression with IL-17 production in PMNs during septic peritonitis and found that both sTLR9 and IL-7 could be expressed in the PMNs infiltrated into the peritoneal cavity of the mice infected with* E. coli*. At early stage of the infection, sTLR9 was increasingly expressed in the infiltrated CD11b^+^ PMNs, and IL-17 was increasingly expressed in both of the CD11b^−^ PMNs and CD11b^+^ PMNs. IL-17 expression in CD11b^−^ PMNs was positively correlated with the doses of* E. coli*. When infected with the highest dose of* E. coli *(2.4 × 10^8^ CFUs), IL-17 was increasingly expressed and sTLR9 was decreasingly expressed in/on the CD11b^−^ PMN. When infected with the lowest dose of* E. coli *(0.8 × 10^8^ CFUs), both IL-17 and sTLR9 were increasingly expressed in CD11b^+^ PMNs. Furthermore, we stained the PLCs with both CD11b mAb and Ly6G mAb and confirmed that the CD11b^+^Ly6G^+^ PMNs in the PLCs could increase their expression of both IL-17 and sTLR9 in response to* E. coli* infection. Taken together, with the fact that highest dose of* E. coli *(2.4 × 10^8^ CFUs) was the most deadly and the lowest dose of* E. coli *was the least deadly to the mice with septic peritonitis, we may deduce that increased expression of both sTLR9 and IL-17 in CD11b^+^ PMNs might benefit the survival of the mice with septic peritonitis and that the decreased expression of sTLR9 and increased expression of IL-17 in CD11b^−^ PMNs may be detrimental to the mice.

In recent years, PMNs have been found to be able to express sTLR9 which engage PAMPs, such as bacterial DNA generated during infection, and damage-associated molecular patterns (DAMPs), such as mitochondrial DNA released from necrotic cells during sterile inflammation. The engagement, if intense, can prime PMNs to release massively produced cytokines [22], leading to sepsis or systemic inflammatory response syndrome (SIRS) [12]. In the present work, we found that the ratios of the sTLR9^+^CD11b^+^ PMNs and sTLR9^+^CD11b^−^ PMNs were significantly increased in the infiltrated CD11b^+^ PMNs and CD11b^−^ PMNs of the mice infected with* E. coli* for 18 hours, respectively. At this stage, we observed that a plenty of* E. coli* coexisted with the PMNs. When observed at 48 hours and 72 hours after infection, the ratios of both of the sTLR9^+^CD11b^+^ PMNs and sTLR9^+^CD11b^−^ PMNs were sharply decreased to the levels as those in saline control. Accompanying with the decrease,* E. coli* underwent disappearing. Possibly, the increased TLR9^+^CD11b^+^ PMNs and sTLR9^+^CD11b^−^ PMNs present a rapid innate immune response of the PMNs to bacterial invasion at the early stage of infection. PMNs armed with sTLR9 were demonstrated to be able to sense extracellular ligands and consequently initiate TLR9 mediated signaling in an intracellular TLR9 independent way. The response could offer a rescue mechanism for PMN activation when pathogen derived TLR9 ligands cannot reach the endosome in the early stage of infection [10]. Practically, the increased expression of sTLR9 on PMNs may be a proinflammatory activation marker [10] and sTLR9^+^ PMN should benefit antibacterial defense during infection, evidenced by the following: (1) TLR9 agonists, if they exist, could activate sTLR9^+^ PMN, not TLR9-deficient PMNs, to upregulate CD11b and secrete MIP-2 and IL-8 (CXCL8) [10]; (2) sTLR9^+^ PMNs are involved in inducing the rapid inflammation which is needed in the initial phase of bacterial infection by secreting antimicrobial peptides and elastases [9]; (3) encountering microbial DNA, sTLR9 signaling is able to activate PMNs, and the activated PMN increases sTLR9 expression [9]. However, it is worthy to note that sTLR9^+^ PMNs could be culprit for causing more severe pathological consequences. For instance, bacterial DNA or formulated peptides released following sepsis were reported to activate p38 MAP kinase through binding sTLR9 on PMNs, leading to acute lung injury which is characterized by protein-rich pulmonary oedema (swelling) and accumulation of large numbers of PMNs in the lungs [13].

It is well established that PMNs, as the firstly recruited inflammatory cells in infection sites, are IL-17 producing cells. IL-17 secreted from PMNs induces the release of proinflammatory factors from mesenchymal and myeloid cells, recruiting additional PMNs [15]. In addition to PMNs, macrophages located in the epithelial barriers are also important sources of IL-17. In this study, we initially found that the average ratios of the IL17^+^CD11b^+^ PMNs and IL17^+^ macrophages were significantly decreased in the PLCs at 18, 48, and 72 hours in the mice infected with 3 doses of* E. coli*. Seemingly, the CD11b^+^ PMNs and macrophages, in response to the peritoneal infection, could not produce increased IL-17. However, when checking mRNA expression, we found significantly upregulated IL-17 mRNA in the PLCs from the mice infected with* E. coli* for 18 hours (Figure 5(e)). The data imply that the infiltrated cells in peritoneal cavity of the mice infected with* E. coli* could produce IL-17 and the produced IL-17 may promptly secrete out the cells, therefore resulting in the decreased ratios of IL17^+^CD11b^+^ PMNs and IL17^+^ macrophages. To clarify this, we harvested the PLCs from the mice infected with* E. coli* at early stage of 8 hours after infection and detected their IL-17 levels which were expressed as mean fluorescence intensity (MFI). By doing this, we found that both of the CD11b^−^ PMNs and CD11b^+^ PMNs obviously produced IL-17 in the early stage of the peritoneal infection caused by* E. coli*. To confirm this, we set up another experiment in which the PLCs were harvested at 12 and 16 hours after the infection, cultured with BFA, a protein transport inhibitor capable of retaining the produced cytokines inside the cells, for additional 4 hours, and then detected their IL-17 expression. We found that the BFA* in vitro* treatment significantly increased the average ratios of the IL17^+^CD11b^+^ PMNs in CD11b^+^ PMNs and IL17^+^ macrophages in macrophages (data not shown). The results support the assumption that produced IL-17 in CD11b^+^ PMNs and macrophages could be secreted out promptly in response to* E. coli *at the early stage of septic peritonitis in mice. Theoretically, deducing based on the published data, IL-17 derived from CD11b^+^ PMNs and macrophages may initiate a response to induce chemokines which in turn recruit more PMNs [23, 24] to the site of infection [18, 25]. The new comer PMNs join the fighting against invaded bacteria, resulting in more IL-17 [16, 17]. In addition to the PMNs, macrophages could be recruited by IL-17 [20, 26], specifically by CD11b^+^ PMNs derived IL-17. IL-17 could recruit more macrophages into peritoneal cavity during septic peritonitis. The increased macrophages could produce more IL-17 to join the CD11b^+^ PMNs produced IL-17, playing a defense role in fighting bacterial infection and reducing mortality of mice with septic peritonitis [27]. In addition to the PMNs and macrophages, *γδ* T cells were found as a major producer of IL-17 in the mice with experimental sepsis induced by CLP; *γδ* T cell-derived IL-17 could promote production of proinflammatory mediators, resulting in enhanced lethality [28]. Compatibly, T cells, in this study, were confirmed to be involved in the development of septic peritonitis in mice by the evidence that (1) the IL-17 expression was increased in the CD3^+^ cells of the PLCs from the mice infected with* E. coli* and (2) IL-17 mRNA levels were significantly increased in the sorted CD3^+^ cells in the PLCs from the mice infected with* E. coli*. Furthermore, IL-17 expression was obviously increased in the sorted CD3^−^CD11b^+^ PMNs in the PLCs from the mice infected with* E. coli*. Together, these data suggest that the PMNs, like T cells, do express IL-17 as a response to bacterial infection during septic peritonitis. Although CD3^+^ cells were one of the major producers of IL-17 during the development of septic peritonitis, probably, neutrophils might produce more IL-17 compared to CD3^+^ cells because the neutrophils, as we found in this study, could constitute up to 70% of the PLCs (Figure 3(b)), whereas CD3^+^ cells only constituted 10–15% of the PLCs (data not shown). Interestingly, sTLR9^−^CD11b^+^Ly6G^+^ PMNs, not sTLR9^+^CD11b^+^Ly6G^+^ PMNs, were found to be able to increase their IL-17 expression during septic peritonitis.

Interestingly, we found a type of giant cells with increasing numbers in the PLCs from the mice infected with* E. coli* up to 1.6 × 10^8^ CFUs. Morphologically, the giant cells (Figure 2(a)) could be the PMNs with a changed lobulated nucleus located near to cell membrane, being at least 4 times larger than the regular PMNs. Obviously, the giant cells are the newly described cells and have several facets worthy of note. (1) Kinetically, the giant cells only appeared at the early stage of the infection and were not observed in the PLCs of the mice infected for 48 and 72 hours. (2) The giant cells only occurred in the PLCs of the severely infected mice, not in the PLCs of the mice infected with less amount (0.4–0.8 × 10^8^ CFUs) of* E. coli*. (3) The giant cells could constitute 20% of the PLCs. Possibly, the giant cells were developed from the regular PMNs after engulfing a large amount of bacterial pathogens. Biologically, the appearance of the giant cells could be used as a cell marker for signifying a real danger because the giant cells were intimately correlated with the severity of the infection and the death of the mice.
